# Ultrasound imaging tracking of mesenchymal stem cells intracellularly labeled with biosynthetic gas vesicles for treatment of rheumatoid arthritis

**DOI:** 10.7150/thno.66905

**Published:** 2022-02-21

**Authors:** Zheli Gong, Yanni He, Meijun Zhou, Huijuan Xin, Min Pan, Muhammad Fiaz, Hongmei Liu, Fei Yan

**Affiliations:** 1 The Second School of Clinical Medicine, Southern Medical University, Guangzhou 510515, China.; 2 Department of Ultrasound, Institute of Ultrasound in Musculoskeletal Sports Medicine, Guangdong Second Provincial General Hospital, Guangzhou 510317, China.; 3 Department of Ultrasound, Hunan Provincial People's Hospital (The First-affiliated Hospital of Hunan Normal University), Changsha, 410061, China.; 4 Shenzhen Hospital of Guangzhou University of Chinese Medicine, Shenzhen, China.; 5 Department of Radiology, Azra Naheed Medical College, Lahore, Pakistan.; 6 CAS Key Laboratory of Quantitative Engineering Biology, Shenzhen Institute of Synthetic Biology, Shenzhen Institutes of Advanced Technology, Chinese Academy of Sciences, Shenzhen, 518055, China.

**Keywords:** Gas vesicles, Ultrasound, Imaging tracking of cells, Mesenchymal stem cells, Rheumatoid arthritis

## Abstract

**Rationale:** Rheumatoid arthritis (RA) is an autoimmune disease characterized by chronic inflammation and damage to articular tissues that can lead to irreversible joint damage and progressive disability. The multipotent mesenchymal stem cells (MSCs) play an important role in immune disorders and tissue regeneration. However, their immunosuppressive effects and the underlying mechanisms are largely unclear due to the lack of tools for real-time imaging of MSCs *in vivo*. Gas vesicles (GVs) are biosynthetic nanobubbles that are ejected from aquatic microbes, such as bacteria and archaea, and have an excellent ultrasound imaging capacity.

**Methods:** We harvested MSCs from the bone marrow of Sprague Dawley (SD) rats. Then, GVs were synthesized and incubated with MSCs to obtain intracellularly labeled MSCs. We firstly tested the ultrasound imaging of GV@MSCs *in vitro* and *in vivo* and then explored the therapeutic effect of GV@MSCs combined with methotrexate (MTX) in RA rats.

**Results:** These GV@MSCs showed significant contrast-enhanced ultrasound signals without a loss of viability and differentiation capacity. In addition, the GV@MSCs could be imaged in real-time for 5 days using ultrasound both *in vitro* and *in vivo*, making it possible to visually track their migration and homing to the joint cavity from the subcutaneous layer of lateral malleolus joints in the injected RA rats. Furthermore, GV@MSCs significantly enhanced the curative effect of methotrexate (MTX) against RA, resulting in decreased paw thickness, lower arthritis index score, reduced bone erosion and cartilage destruction, compared to the PBS, free MTX, and GV@MSCs groups.

**Conclusion:** We developed a novel therapeutic strategy against RA using GVs-loaded MSCs that can be tracked* in vivo* in real-time.

## Introduction

Rheumatoid arthritis (RA) is a chronic autoimmune disease that is characterized by persistent synovitis, systemic inflammation, and the production of autoantibodies. RA often causes irreversible joint damage, systemic complications and progressive disability 1. It currently affects about 0.24% of adults, worldwide with 20-45 new cases being diagnosed per 100,000 individuals each year, especially among females and the elderly people 2. Uncontrolled active RA results in tissue inflammation that leads to progressive erosive joint damage, disability, and cardiovascular and other comorbidities 3. Current treatment strategies for RA include nonsteroidal anti-inflammatory drugs (NSAIDs), disease-modifying antirheumatic drugs (DMARDs) and biological agents with the primarily aim of suppressing autoimmune inflammations. NSAIDs provide symptomatic relief against pain and swelling 4, whereas DMARDs can reduce synovitis and systemic inflammation, inhibit joints damage and improve joint function 5. Methotrexate (MTX) is a DMARD that is widely used in a clinical setting but the clinical dose used produces harmful side-effects, such as gastrointestinal reaction, hepatorenal toxicity, pulmonary toxicity and immunosuppression. Biological agents, such as tumor necrosis factor (TNF) or interleukin-6 (IL-6) receptor inhibitors, can target the inflammatory cytokines and pathways that cause tissue damage 6, 7. Novel studies used PTT and PDT therapies against hyperplastic synovial tissue were conducted recently and were shown to exert a remarkable therapeutic effect 8, 9. Although the outcomes of therapy for RA have improved significantly in recent years, remission is possible for many patients, while many others do not show a satisfactory response 10. Cartilage and bone damage may persist even after clinical remission has been achieved. Therefore, it is essential to develop novel treatment strategies that induce remission through permanent immune tolerance, and repair and prevent structural erosion.

Mesenchymal stem cells (MSCs) are multipotent cells that can exert immunomodulatory and tissue regenerative functions. Studies have shown that MSCs regulate both adaptive and innate immune responses through multiple mechanisms, such as the production of immunosuppressive or inflammatory cytokines 11, 12, macrophage polarization to the anti-inflammatory phenotype 13, recruitment of MSCs or other lymphocytes through chemokine secretion 14-16, and T-cell inhibition via nitric oxide (NO) 17. Furthermore, MSCs can exert their effects through direct cell-cell contact, as well as in a paracrine manner via the secretion of soluble factors, such as prostaglandin E2, indoleamine 2,3-dioxygenase (IDO) and interleukin (IL)-10 18. Given their ability to attenuate the pathological immune response, MSCs are a promising therapeutic tool against autoimmune diseases, including RA 19. Clinical studies have shown a satisfactory safety profile of allogeneic MSCs in RA patients. However, the mechanisms that underlie their immunosuppressive functions are largely unknown, primarily due to a lack of suitable imaging tools for tracking MSCs in real-time.

Optical imaging, magnetic resonance imaging (MRI), and radionuclide scanning are currently used to track MSCs that are labelled with suitable probes 20-22. Optical imaging has high spatial and temporal resolution but limited tissue penetration depth and risk of radiation exposure. Although MRI has high spatial resolution, soft tissue contrast and depth of tissue penetration 23-25, its temporal resolution is limited. Ultrasound has several advantages over other imaging modalities, including safety, low cost, high tissue penetration depth, excellent spatial resolution, and real-time imaging. MSCs labelled with microbubbles (MBs) may generate ultrasound contrast signals when ligand-coated microbubbles attach to the surface of the receptor and are retained on it for the duration time of imaging, which makes the targets detectable *in vivo*. However, since microscale MBs are too large to enter cells and collapse in circulation 26-28, the imaging duration of these externally labeled MSCs lasts only minutes, which limits their use for *in vivo* cell tracking 29-31.

**Figure 1:**
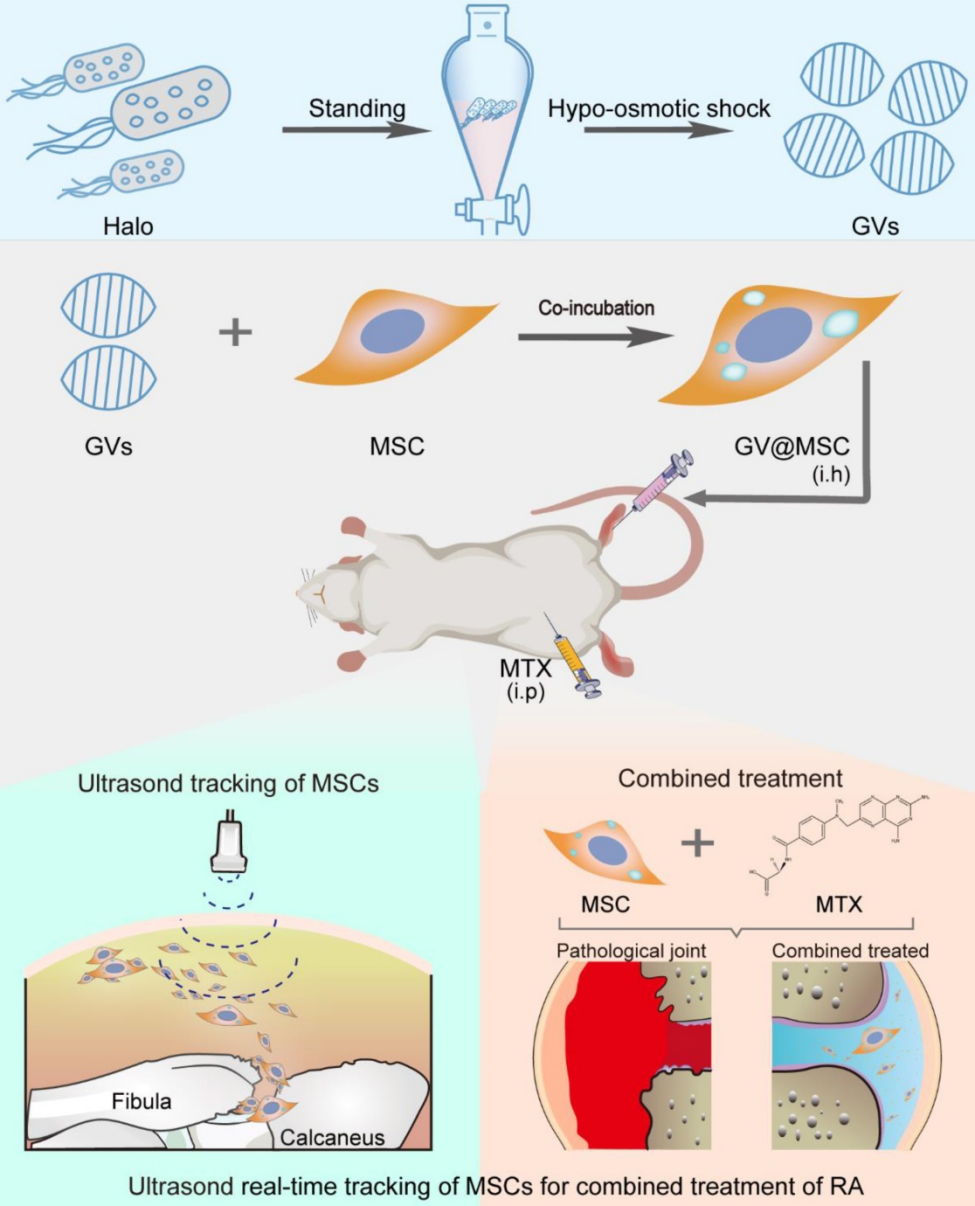
Schematics of real-time imaging and combination treatment using GV@MSCs. The GVs were isolated from the archaeon *Halobacterium* NRC-1 (Halo) and loaded into the MSCs. The GV@MSCs were subcutaneously transplanted into the lateral malleolus joints of arthritic rats, and their migration and homing movement were tracked in real-time using ultrasound. The accumulation of GV@MSCs at the diseased site mitigated inflammation, and protects bones and cartilage. In addition, the GV@MSCs augmented the therapeutic effects of MTX.

Gas vesicles (GVs) are nanoscale particles (~200 nm) that contain gas encapsulated within 2 nm-thick protein shells, and are produced by aquatic bacteria and archaea 32, 33. GVs function as flotation devices that allow aquatic microbes to reside at a suitable depth in the aqueous environment depending on the relative intra-vesicular gas content 34. GVs are biocompatible and show excellent ultrasound imaging capacity 35, 36. Therefore, GVs can be developed as ultrasound contrast agents for intracellular labelling and tracking of MSCs. In this study, we isolated GVs from *Halobacterium* and loaded them into rat MSCs, and transplanted the GV@MSCs into arthritic rats. Their migration and homing movement to the diseased site can be visually tracked in a real-time manner through ultrasound imaging. Furthermore, the combination of GV@MSCs and MTX, a widely used drug in a clinical setting to treat RA, which significantly improves the therapeutic outcome (Figure 1).

## Materials and methods

### Preparation of GVs

GVs were isolated from the bacterial culture, as previously described 32, 35. In brief, the archaeon *Halobacterium* NRC-1 (Halo) was cultured at 42 °C in ATCC medium with constant shaking at 100 rpm for 2 weeks. The culture broth was transferred into a separatory funnel and was left undisturbed for a week to allow the buoyant cells to produce GVs and float to the top. The floating bacteria were separated from the media and then lysed using a series of hypo-osmotic shocks. Then, the GVs were isolated from the lysates using floatation centrifugation 3 times at 300 g for 4 h, and were resuspended in deionized water.

### MSCs isolation and culture

The MSCs were harvested from the bone marrow of two-weeks-old male Sprague Dawley (SD) rats (Vital River Laboratory Animal Technology, Beijing, China) as previously described 37. In brief, the femurs and tibias were extracted under sterile conditions and their ends were cut. The marrow was flushed out using F12 Dulbecco's Modified Eagle's Medium (DMEM/F12; Gibco) supplemented with 10% fetal bovine serum (FBS) and 1% penicillin/streptomycin. Single cells were obtained by passing the suspension through a 70-mm filter mesh, and were then incubated at 37 °C in a humidified chamber with a 5% CO_2_ atmosphere. The non-adherent cells were removed 3 h later by aspirating the media and fresh media was added. The culture media was replaced every 8 h. After 72 h of culture, the primary MSCs were passaged and the cells were expanded for 3-4 passages before being used in the other experiments.

### Cytotoxicity assay

MSCs were seeded into 96-well plates at a density of 3 × 10^4^ cells per well and cultured for 12 h in the presence of different doses of GVs (OD_500_ = 0.1, 0.25, 0.5, 0.75 and 1) for varying durations (1, 2, 4, 6, 8, 10,12, 24 and 48 h). Cell viability was determined by performing CCK-8 assay (Dojindo Laboratories, Tokyo, Japan) to determine the optimum GVs concentration and incubation time. The viability of the GV@MSCs was also assessed after 1, 2, 3, 4 and 5 days of culture by performing CCK-8 assay, while the absorbance at 450 nm was measured using a multimode plate reader (SynergyTM4, BioTek, VT, USA). The treated cells were stained using a Calcein-AM/PI cell viability kit (Beyotime Institute of Biotechnology, China), and was counted under a light microscope to calculate the number of viable and dead cells.

### Preparation and Characterization of GV@MSCs

1, 1'-dioctadecyl-3, 3, 3', 3'-tetramethylindocarbocyanine perchlorate (DiI, Beyotime Institute of Biotechnology, China) dye was incubated with the GVs to obtain the DiI-stained GVs. The MSCs were incubated with DiI-stained GVs (OD_500_ = 0.75) for 10 h, and washed with PBS to remove the free GVs. Then, the fluorescence labeled GV@MSCs were stained with FITC-conjugated wheat germ agglutinin (WGA-FITC, Sigma-Aldrich, St Louis, MO, USA) and DAPI (Beyotime Institute of Biotechnology, China), and their phagocytic ability was evaluated using a confocal laser scanning microscope (A1R, Nikon, Japan) and multimode plate reader. The morphology and subcellular localization of the GVs in the MSCs was further ascertained using a transmission electron microscope (JEM-1230, JEOL, Japan).

### Cell migration assay

The GV@MSCs or MSCs were seeded into the upper chambers of Transwell inserts (24-well insert, pore size 8 μm) at a density of 1 × 10^3^ cells per well in serum-free DMEM. The lower chambers were filled with complete DMEM with or without SDF-1α (200 ng/mL, Sigma-Aldrich, St. Louis, MO, USA). After 12 h of incubation, the cells that had migrated to the lower side of the Transwell were stained with crystal violet, and counted under a light microscope to calculate the migration rate.

### *In vitro* MSCs differentiation

The GV@MSCs and MSCs were subjected to adipogenic, osteogenic and chondrogenic differentiation using an induction media specific for them with or without TNF-α (200 ng/mL, Sigma-Aldrich, St. Louis, MO, USA) supplementation, as instructed by the manufacturer (Cyagen Biosciences, Guangzhou, China). Oil red staining was used to stain the fat droplets after 22 days of adipogenic induction. Alizarin red and Alixin blue were respectively used to stain calcium deposits and chondrocytes after 28 days of osteogenic and chondrogenic induction.

### *In vitro* ultrasound imaging of GV@MSCs

The ultrasound contrast-enhanced ability of the GV@MSCs (1 × 10^5^, 1 × 10^6^, 1 × 10^7^, 1 × 10^8^, 1 × 10^9^ cells) and their imaging duration were examined using a high frequency ultrasound imaging system (Vevo 2100, Visual Sonics, Toronto, Canada) equipped with a MS 250 probe. MSCs with similar cellular densities were used as controls. The GV@MSCs and MSCs were added into phantom holes containing 3% (w/v) agarose powder (VetecTM, MC, USA). Images were captured in B-mode and contrast mode, while all imaging parameters (frequency: 18 Hz; transmit power: 4%; dynamic range: 35 dB) were kept constant. Echo signal intensities were analyzed using VevoCQ 1.3.12.0 analysis software on the Vevo 2100 imaging platform.

### Establishment of a collagen-induced arthritis (CIA) rat model and treatment regimen

The MSCs were isolated from the bone marrow of two-weeks-old male Sprague Dawley (SD) rats, as previously described. Adult female SD rats weighing 180-200 g were used to establish a collagen-induced arthritis (CIA) model. In brief, bovine type II collagen (CII, Chondrex, USA) in 0.1 M acetic acid was emulsified with equal volumes of complete Freund's adjuvant (CFA, Chondrex, USA) containing 2 mg/mL *Mycobacterium tuberculosis*. Each animal was subcutaneously injected at the base of the tail with 100 μL of the emulsion, and a booster injection of CII emulsified in incomplete Freund's adjuvant (IFA, Chondrex, USA) was administered 7 days later. The hind paw thickness and arthritis index (AI) were evaluated every 2 days. The AI was graded from 0 to 3, as previously described 38, and a score of 2 was indicative of successful CIA modelling. The twenty-four CIA rats were divided into the following treatment groups: PBS (saline), MTX (intraperitoneal injection of 2 mg/kg MTX), GV@MSCs (subcutaneous injection of GV@MSCs), GV@MSCs + MTX (subcutaneous injection of GV@MSCs and intraperitoneal injection of 1 mg/kg MTX). All treatments were repeated every 7 days over a period of four weeks. Six healthy rats served as the normal control. The rats were observed every other day, and euthanized 38 days after the first immunization. Ankle joints and blood samples were obtained for follow-up experiments. All animal experiments were approved by Shenzhen Institutes of Advanced Technology and the Animal Care and Use Committee of the Chinese Academy of Sciences.

### *In vivo* ultrasound imaging of GV@MSCs

The arthritic rats were subcutaneously injected with 1 × 10^7^ GV@MSCs or MSCs into their lateral malleolus. The B-mode and contrast-enhanced ultrasound images were captured every day for 5 days after injection. The imaging parameters used are as stated above.

### ELISA

Blood was collected from each animal, and the serum fraction was isolated. TNF-α levels were measured using an ELISA kit, by following the manufacturer's instructions (Boster Biological Technology, California, USA). The optical density (OD) was read at 450 nm on a microplate reader.

### Radiological and histological assessment of ankle joints

High-resolution micro-computer tomography (Micro CT) was used for the 3D reconstruction of the ankle joints (SkyScan 1176, Bruker, GER). The processing parameters were as follows: number of layers = 57, pixel size = 18.09 μm, lower grey threshold = 103 and upper grey threshold = 255. The morphometric parameters of the trabecular bone, including bone volume fraction (BV/TV %) and the mean bone mineral density (BMD) of the talus, were calculated using CTAn software (Skyscan). MRI was performed (Bruker Biospin Advance 9.4 T (400 MHz) system, transmit-receive quadrature coil) to obtain representative images of the mid-sagittal planes of each ankle (Analyze 10.0, Mayo Clinic, Rochester). The ankle joints of the hind paws were harvested after imaging, and were sectioned for H&E and safranin O-fast green staining. The immunohistochemical staining of TNF-α was also performed, following standard protocols.

### Statistical analysis

All data are expressed as mean ± standard error. Independent *t*-test was used to compare two groups. While multiple groups were compared using one-way ANOVA analysis followed by Tukey's post-test. The weights of each different group were compared using a mixed linear model. SPSS 22.0 software (SPSS, Chicago, IL, USA) was used for all analyses and *p* value of < 0.05 was considered to indicate statistical significance.

## Results and Discussion

### Fabrication and characterization of the GV@MSCs

**Figure 2:**
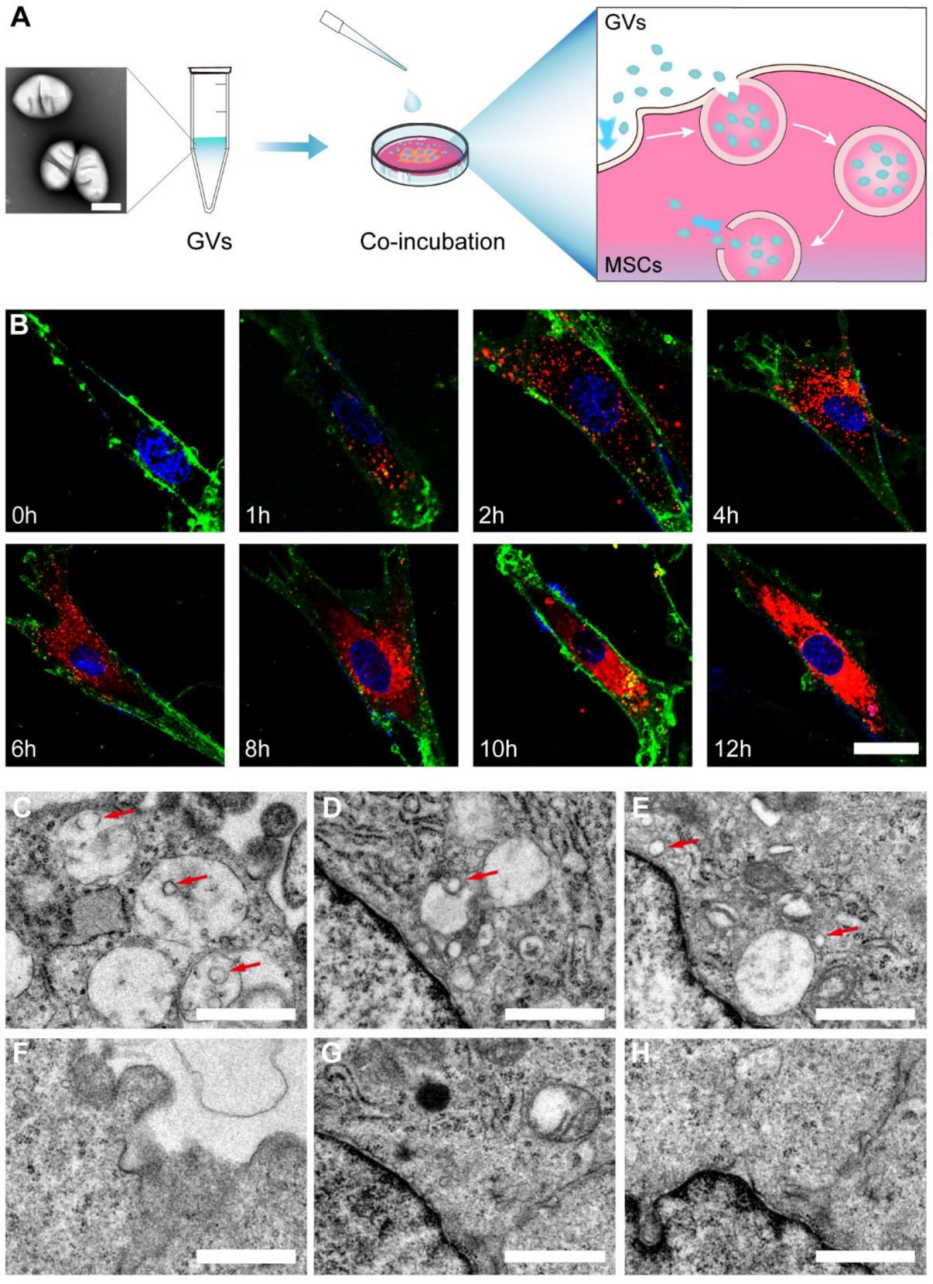
** The preparation and characterization of GV@MSCs. (A)** Schematic illustration of GV@MSCs synthesis. **(B)** Confocal images showing time-dependent phagocytosis of DiI-labeled GV@MSCs by the MSCs. The nuclei were stained with DAPI (blue) and the cell membranes with FITC-conjugated wheat germ agglutinin (green). Scale bar = 3 µm. **(C-H)** TEM images of the GV@MSCs (C-E) and MSCs (F-E) showing the internalization of GVs into the MSCs through endosomes, followed by cytoplasmic release. Red arrows are used to indicate the GVs. Scale bar = 1 µm.

The GVs were isolated from *Halobacterium* NRC-1 (Halo) cells using hypo-osmotic cell lysis and were purified using centrifugal floatation (Figure S1) 36. Transmission electron microscopy (TEM) revealed that the GVs had an olive-shaped morphology and core/shell structure (Figure S2). The average hydrodynamic diameter of the GVs as measured using dynamic light scattering (DLS) was 218.43 ± 17.38 nm, with a polydispersity index (PDI) of 0.19 ± 0.04 (Figure S3). The zeta potential of the GVs was about -8.47 ± 0.15 mV. The flow cytometric analysis of the harvested MSCs revealed positive expression of CD29 and CD90, and negative expression of CD45 and CD106 (Figure S4). Following incubation with MSCs, the isolated GVs were loaded into the cells through phagocytosis. As shown in Figure 2B, S5 the number of Dil-labeled GVs phagocytosed into the MSCs gradually increased in a time-dependent manner. The viability of the MSCs was not significantly affected by the tested concentrations of GVs (Figure S7), indicating excellent biocompatibility. Bio-TEM indicated the presence of GVs in the GV@MSCs but not in the control MSCs (Figure 2C-H). In addition, the GVs were observed in the endosomes (Figure S6), which indicated that the phagocytosis of GVs proceeded through the endocytic membrane transport pathway. Then, we calculated the number of GVs in MSCs as shown in the TEM images and found an average of 39 GVs per MSC.

### GVs have no effect on the viability and functions of the MSCs

**Figure 3:**
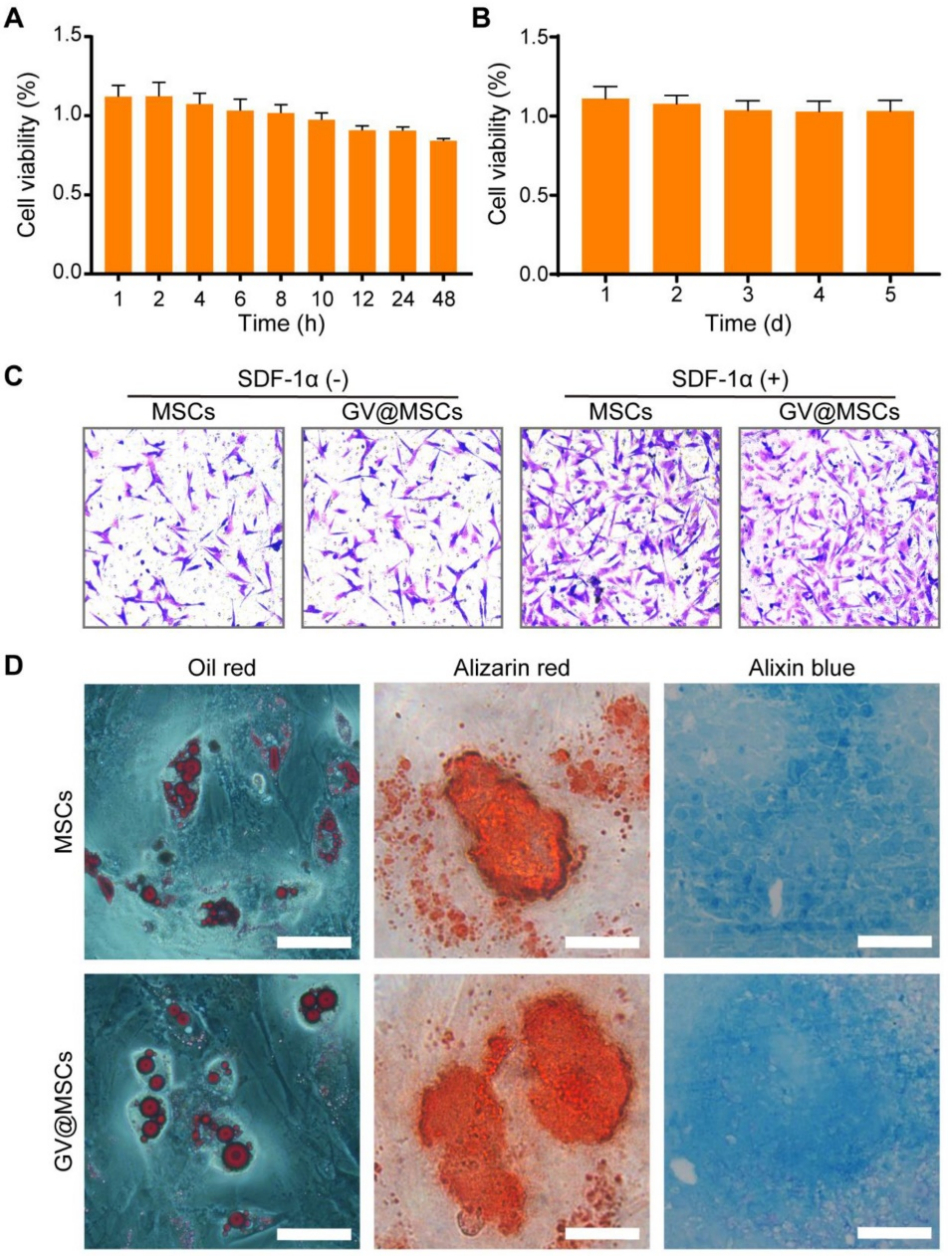
** Viability and differentiation of GV@MSCs.** The concentration of the GVs was OD_500_ = 0.75. **(A)** Percentages of viable MSCs incubated with the GVs for 1, 2, 4, 6, 8, 10, 12, 24, and 48 h at 37 °C (5 replicates each). **(B)** Percentages of viable GV@MSCs after incubation for 5 days. The free GVs were removed after 10 h (5 replicates). **(C)** Representative images of MSCs and GV@MSCs that had migrated to the lower Transwell chamber after 12 h in the presence or absence of SDF-1α. Scale bar = 150 µm (n = 15 fields). **(D)** Representative images of Oil red, Alizarin red, and Alixin red stained MSCs and GV@MSCs, indicating the presence of adipocytes, osteoblasts and cartilage. Scale bar = 50 µm.

The MSCs were incubated with GVs (OD_500_ = 0.75) for varying durations to determine their potential cytotoxicity. As shown in Figure 3A, there was no significant decrease in the percentage of viable MSCs after 48 h of incubation with the GVs. In addition, live and dead double staining assay of the MSCs co-incubated with GVs (OD_500_ = 0.75) did not show an increase in the number of dead cells, compared with the control MSCs (Figure S8). Even prolonged exposure to the GVs for 5 days did not affect the viability of MSCs (Figure 3B), indicating that the GVs are non-toxic and can be used to label cells. MSCs demonstrated a homing action and accumulate in the inflamed lesions, and secrete anti-inflammatory factors as well as chemokines to recruit endogenous stem cells 39-42. The potential impact of the GVs (OD_500_ = 0.75) on the migration of MSCs was evaluated by performing Transwell assay. No significant difference was observed in the migration capacity of the GV@MSCs and MSCs, indicating that the GVs did not affect the homing behavior of MSCs (Figure 3C, left). High levels of SDF-1α in the arthritic joints promoted the migration of the MSCs relative to that observed in healthy joints 43, 44. Next, we analyzed the migratory capacity of the MSCs in the presence of SDF-1α. As expected, SDF-1α significantly increased the number of GV@MSCs and MSCs that migrated to the bottom of the Transwell chamber, while no significant difference was observed between the two groups (Figure 3C, S9). The GV@MSCs and unlabeled MSCs were cultivated in adipogenic, osteogenic, and chondrogenic differentiation media, and the results of the respective staining assays conducted to determine the presence of fat droplets, calcium deposits, and chondrocytes indicated that the GVs did not affect the differentiation capacity of the MSCs (Figure 3D, S10). The GV@MSCs used in the above experiments were fabricated using the GVs (OD_500_ = 0.75) co-incubated with MSCs for 10 h.

### Ultrasound imaging properties of the GV@MSCs

**Figure 4:**
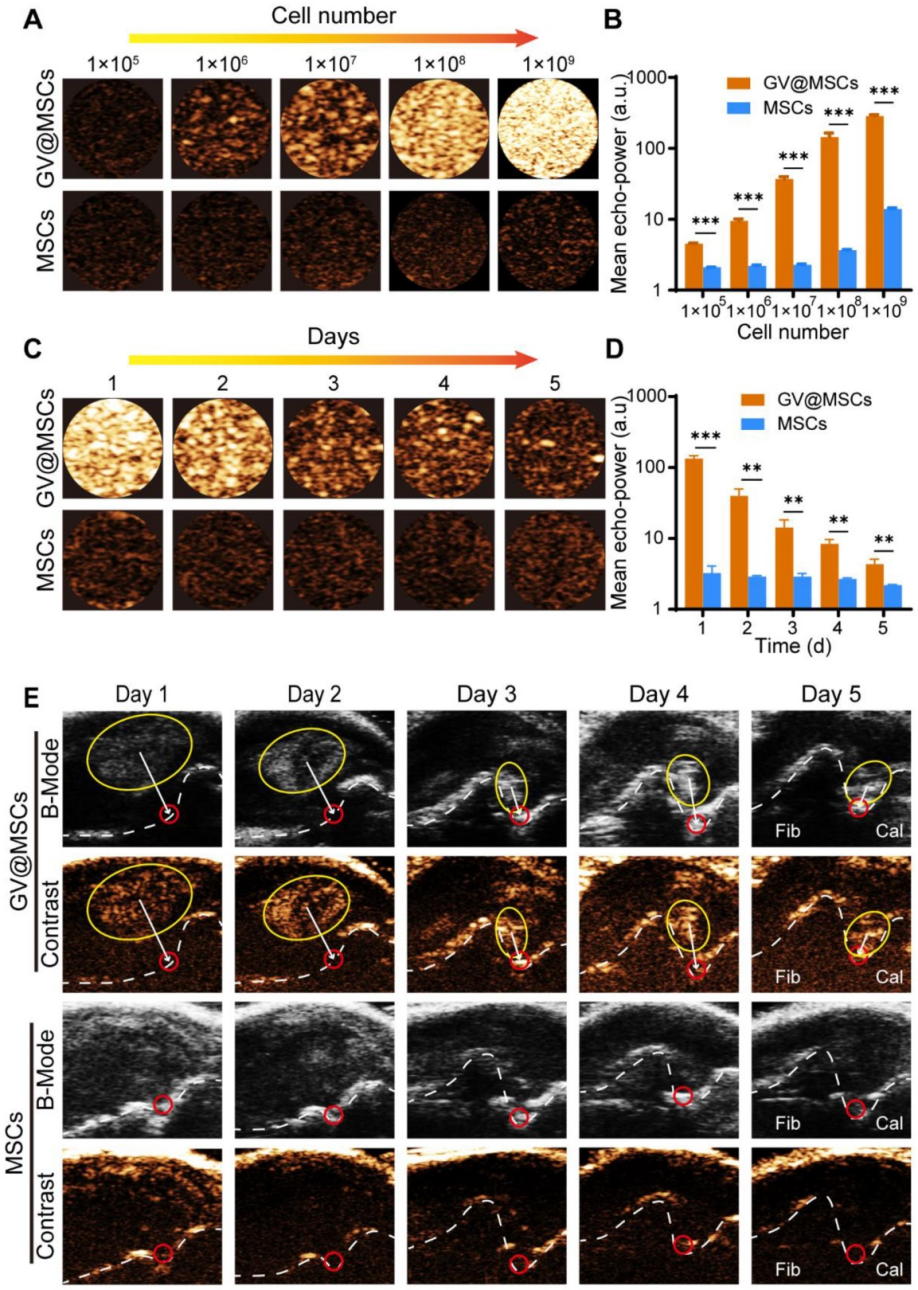
** Ultrasound imaging of the GV@MSCs *in vitro* and real-time imaging tracking of the GV@MSCs *in vivo*. (A)** *In vitro* contrast enhanced ultrasound images and **(B)** echo signal intensity of the MSCs or GV@MSCs at different concentrations (performed in triplicate). **(C)** *In vitro* contrast enhanced ultrasound images and **(D)** echo signal intensity for different durations (performed in triplicate). **(E)** *In vivo* real-time tracking of GV@MSCs injected subcutaneously injected into the lateral malleolus joints of CIA model rats in B-mode and contrast mode (performed in triplicate) (Fib: Fibula; Cal: Calcaneus). **, *p* < 0.01. ***, *p* < 0.001.

Several studies have demonstrated the excellent ultrasound imaging capability of the GVs 35, 45. However, the critical collapse pressure of GVs from Halo is 70-150 kPa 36, which makes GVs easily collapsible and probable to lose the echo signal when injected directly. To test the ultrasound contrast properties of the GV@MSCs, we suspended a varying number of GV@MSCs and MSCs in PBS. The GV@MSCs displayed enhanced acoustic signals and the contrast increased in a concentration-dependent manner, with the maximum contrast being observed with 1 × 10^9^ cells. In contrast, the signals intensity of the MSCs did not change significantly along with the increase in cell numbers (Figure 4A). Quantitatively, the GV@MSCs showed a 2.13-, 4.32-, 16.15-, 39.25- and 20.44-fold higher acoustic signal intensities with 1 × 10^5^, 1 × 10^6^, 1 × 10^7^, 1 × 10^8^ and 1 × 10^9^ MSCs, respectively (Figure 4B). Considering the need for the long-time tracking of MSCs for therapeutic applications, we determined the imaging duration of the GV@MSCs *in vitro*, and found that the signals persisted for 5 days (Figure 4C). Although the contrast signals diminished along with time, the signal intensities of the GV@MSCs were 41.00-,13.75-, 4.92-, 3.13-, and 1.97-fold higher compared to that of MSCs after 1, 2, 3, 4, and 5 days, respectively (Figure 4D). To determine *in vivo* ultrasound contrast imaging and migration of the GV@MSCs, 1 × 10^7^ GV@MSCs or MSCs were subcutaneously injected into the lateral malleolus joints of a rat CIA model and ultrasound images were obtained in the B-mode and contrast mode for 5 days. Strong enhanced acoustic signals from the GV@MSCs were detected immediately after transplantation, which showed that they had gradually migrated towards the arthritic ankle joints and were mainly concentrated in the joint cavity after 5 days (Figure 4E, red circles). A short high energy burst could quickly eliminate these acoustic signals from GV@MSCs due to GVs collapse in MSCs (Figure S11). In contrast, no acoustic signals from the transplanted MSCs were observed in either B-mode or contrast mode. Thus, it is possible to track the GV@MSCs but not the MSCs using real-time ultrasound imaging. Furthermore, the ankle joints of the animals treated with GV@MSCs showed numerous MSCs that positively expressed of CD29.

### Combination therapy using GV@MSCs and MTX for RA *in vivo*

**Figure 5:**
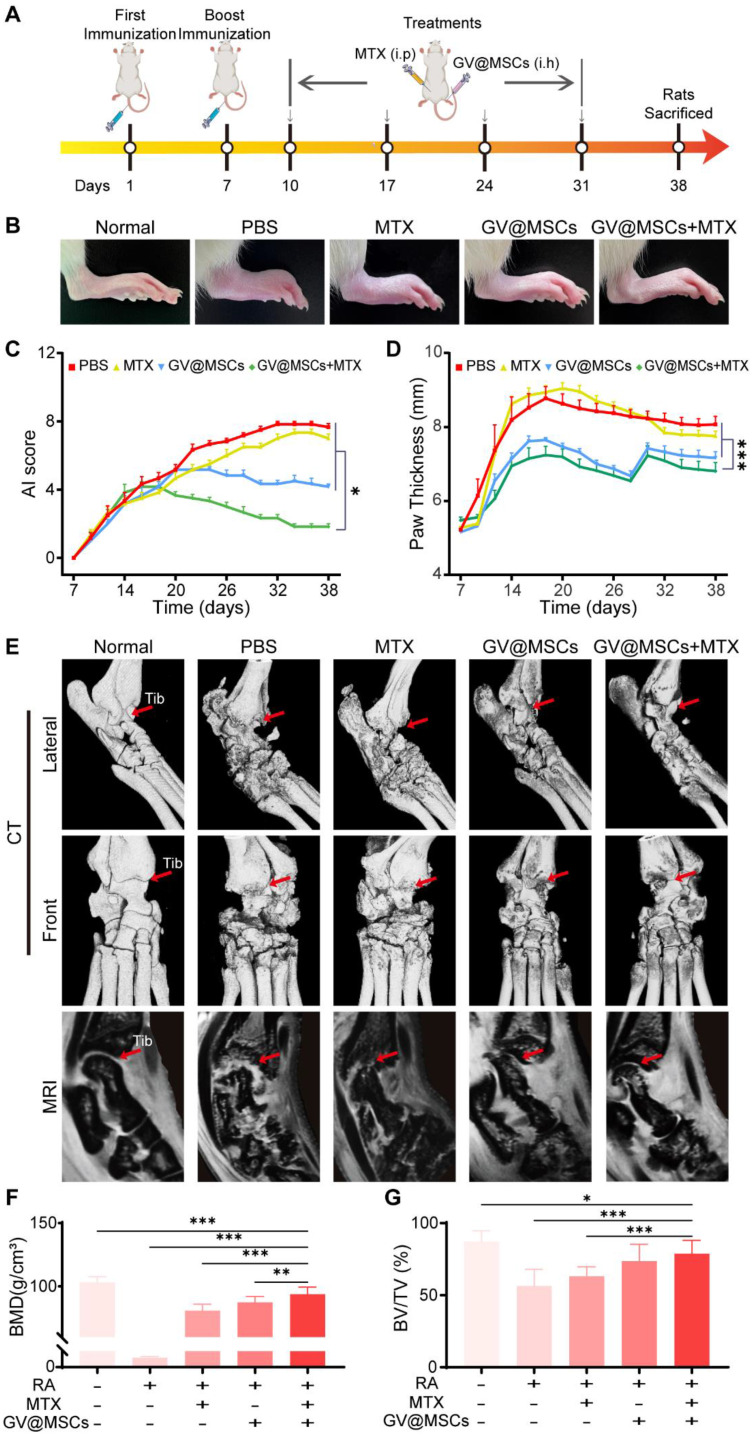
***In vivo* therapeutic efficacy of GV@MSCs and MTX against RA. (A)** Treatment protocols for the CIA rat model. The animals were subcutaneously injected four times with GV@MSCs with or without MTX. **(B)** Macroscopic images of the ankle tissues of normal and CIA rats after 38 days (n = 5) of the indicated treatments. **(C and D)** Changes in paw thickness and AI scores in the different treatment groups. **(E)** Micro-CT 3D reconstruction and MRI images of joint structures on day 38 (red arrow indicates the tibiotalar joint, Tib: Tibiotalar). **(F and G)** BMD and BV/TV of the different treatment groups calculated using the CT images. *, *p* < 0.05. **, *p* < 0.01. ***, *p* < 0.001.

The *in vivo* therapeutic efficacy of the GV@MSCs with or without MTX was evaluated using a CIA model. The treatment scheme used is shown in Figure 5A. These CIA rats were randomly divided into four groups and injected with PBS, free MTX, GV@MSCs or GV@MSCs + MTX (half dose), while healthy rats were used as the control. MTX was administrated through intraperitoneal injection and GV@MSCs were subcutaneously injected into the ankle to simulate the clinical administration route. Joint damage was measured by monitoring the mean AI score and hind paw thickness. As shown in Figure 5B-D, the joints of heathy rats were normally shaped and their paws did not show any redness or swelling. In contrast, CIA model rats with PBS or only MTX showed an obvious redness and swelling of paws, high AI score and increased paw thickness. Injection of GV@MSCs significantly relieved redness and swelling, and reduced the AI score and paw thickness. The combination of GV@MSCs + MTX showed the strongest therapeutic efficacy, with minimal redness and swelling of paws, the lowest AI score and no thickening of paws.

We also analyzed the damage to the bone and cartilage using micro-CT 3D reconstruction and MRI. As shown in Figure 5E, the normal rats showed healthy joint structure without cortical erosion or bone destruction. On the other hand, CIA rats had obviously disordered ankle joints, with apparent cortical erosion and bone hyperplasia. Although, free MTX and GV@MSCs partially alleviated the symptoms of arthritis, the combination of both resulted in almost complete recovery with only mild bone and cartilage destruction (Figure 5E). MRI data clearly revealed that the articular surface and cartilage layer in the GV@MSCs + MTX group was relatively more uniform, as opposed to the visible erosion seen in the PBS, MTX and GV@MSCs groups (Figure 5E). Quantitative analyses of trabecular bone damage levels from the Micro-CT images showed the lowest bone mineral density (BMD) and BV/TV ratio in the PBS group. Slight recovery of both was observed in the free MTX group, whereas GV@MSCs and GV@MSCs + MTX provided maximum recovery, indicating that the MSCs exert a protective effect on the bone and cartilage (Figure 5F, G).

### Histopathological analysis

**Figure 6:**
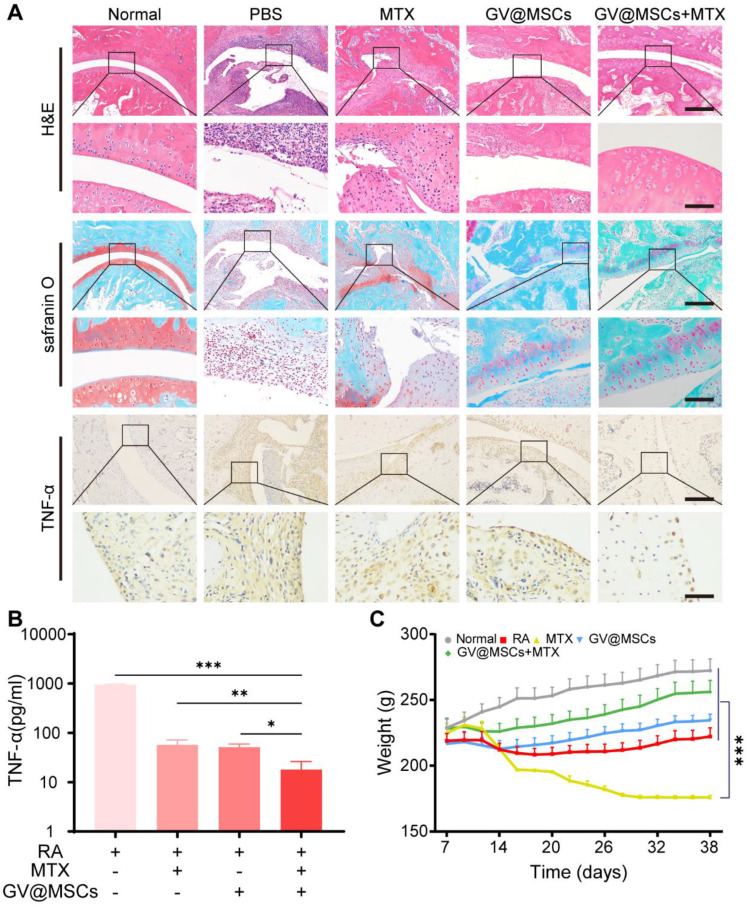
** Histological analysis. (A)** H&E staining (upper) and Safranin O-fast green staining (middle) of ankle joint sections. Scale bar = 200 µm. Immunohistochemical staining of TNF-α in ankle joint sections (lower). Scale bar = 200 µm. **(B)** TNF-α levels in the serum. **(C)** Body weight of rats in the different treatment groups. *, *p* < 0.05. **, *p* < 0.01. ***, *p* < 0.001.

H&E staining of the ankle joints sections obtained from the untreated arthritic animals showed massive infiltration of inflammatory cells into the articular cavity, and obvious bone erosion in the inflamed joints. In contrast, MTX and MSCs monotherapies decreased inflammation and bone erosion. The combination of GV@MSCs and MTX markedly decreased the infiltration of inflammatory cells, resulting in a nearly normal articular cavity surface. Safranin O-fast green staining assay showed the least proteoglycan loss in the GV@MSCs + MTX group, indicating minimum damage to the cartilage (Figure 6A). Furthermore, different treatments markedly decreased levels of the pro-inflammatory cytokine, TNF-α, in ankle joint tissue compared with the untreated control. The serum levels of TNF-α in rats treated with MTX, MSCs or GV@MSCs + MTX were 16.47-, 18.53- and 52.37-fold less, compared with those in the PBS group (Figure 6A, C).

MTX is associated with numerous side-effects due to the relatively large dosage required. In our study, rats treated with free MTX were morbid and showed significant weight loss, poor activity, diarrhea and a high mortality rate (Figure 6B). Only 2 rats in this group survived after the 38-day treatment regimen. In contrast, the rats treated with GV@MSCs + MTX were in a better state and had a higher body weight, compared with rats in the other groups. Taken together, our results indicate that the GV@MSCs improved the therapeutic efficacy of MTX against RA.

## Conclusions

We loaded MSCs with nanoscale GVs to treat RA. Owing to the natural biocompatibility of GVs, the GV@MSCs were viable and retained their differentiation capacity. In addition, the GV@MSCs could be tracked *in vivo* over a period of 5 days in real-time using ultrasound imaging. The combination treatment using GV@MSCs and MTX resulted in greater immunosuppression and bone/cartilage regeneration, compared to either form of treatment administered as a monotherapy. Overall, our study provides novel insights into the biomedical applications of MSCs.

## Supplementary Material

Supplementary figures.

## Figures and Tables

**Scheme 1:**
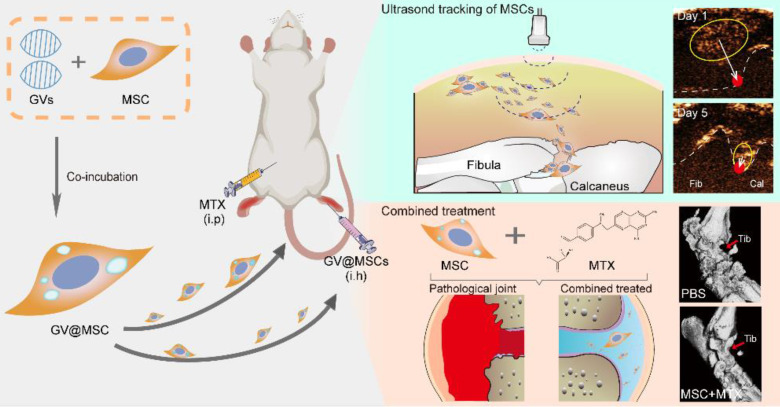
Schematic illustration of ultrasound real-time tracking of MSCs and combined with MTX against RA.
