# Supplementary material for: Ultrasound imaging tracking of mesenchymal stem cells intracellularly labeled with biosynthetic gas vesicles for treatment of rheumatoid arthritis
Source: Theranostics. 2022 Feb 21;12(5):2370–82. doi: 10.7150/thno.66905 (PMC8899585; doi:10.7150/thno.66905)
Supplement: Supplementary file 1 — Supplementary figures. [file thnov12p2370s1.pdf]

Supplementary data

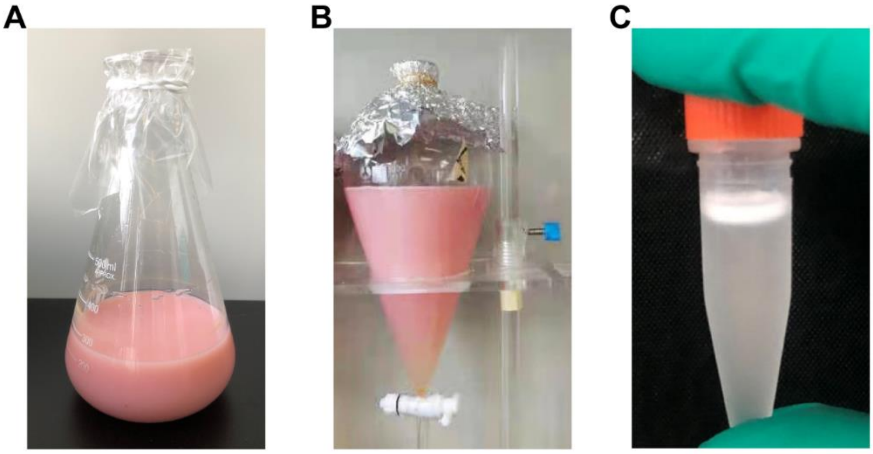

Figure S1. Isolation and purification process of GVs. (A) Pink archaeon *Halobacterium NRC-1* (Halo) before harvest. (B) Separation of buoyant bacterial cells in a separatory funnel. (C) The GVs were isolated from bacteria and floated on the top of the media.

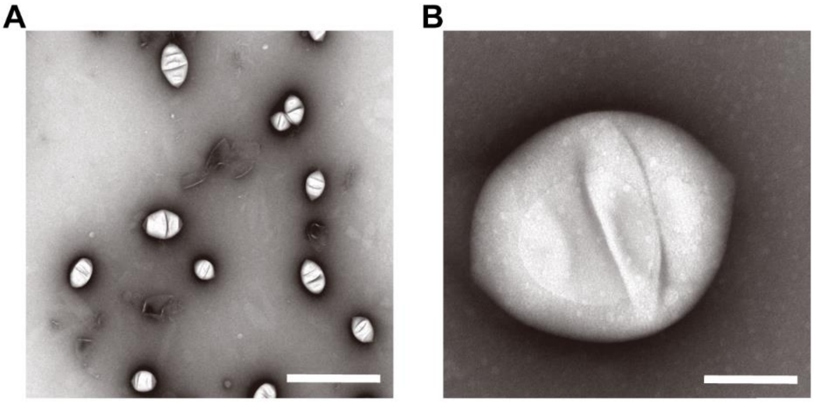

Figure S2. TEM image of GVs. (A) Scale bar = 1μm (B) Scale bar = 200 nm.

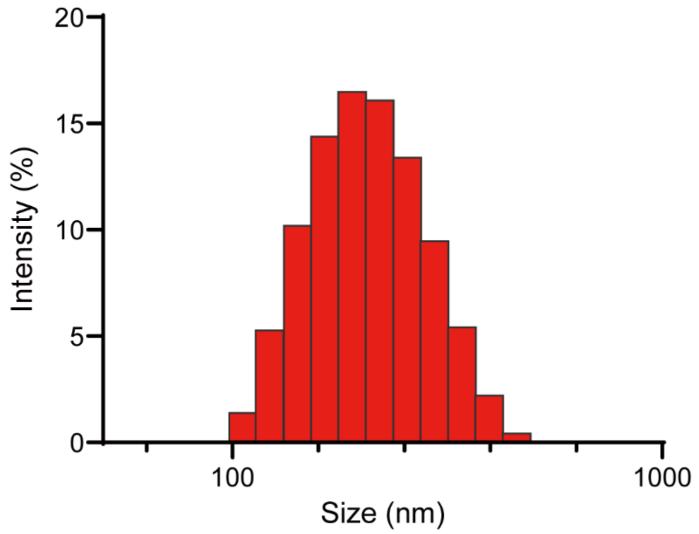

Figure S3. Size distribution of GVs by dynamic light scattering.

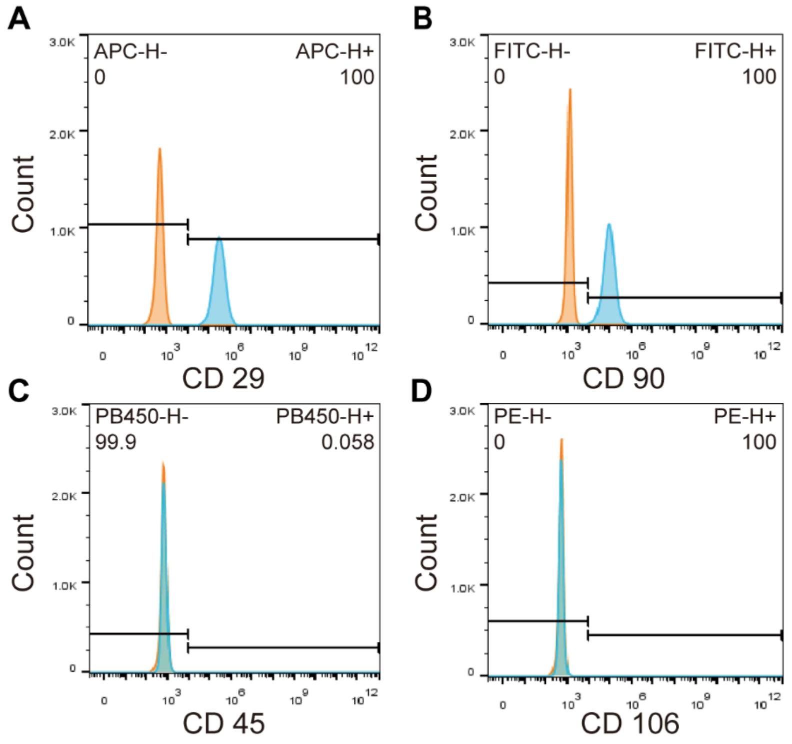

Figure S4. Flow cytometric analysis of harvested MSCs. The MSC populations revealed positive expression of CD29, CD90, and negative expression of CD45, CD106.

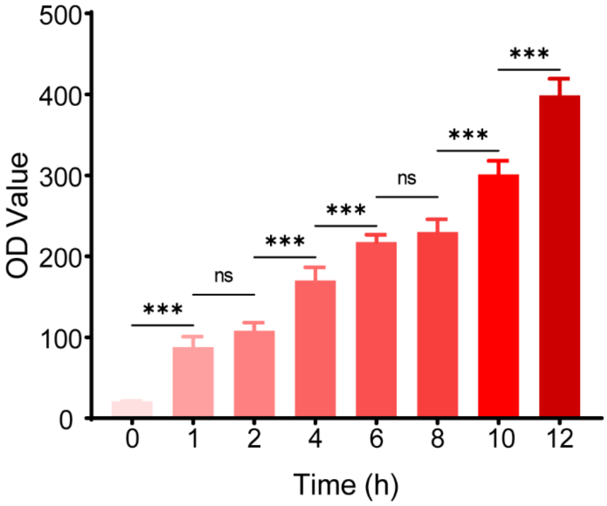

Figure S5. Quantitative analysis of GV@MSCs after being incubated for different time. ***, $p < 0.001$

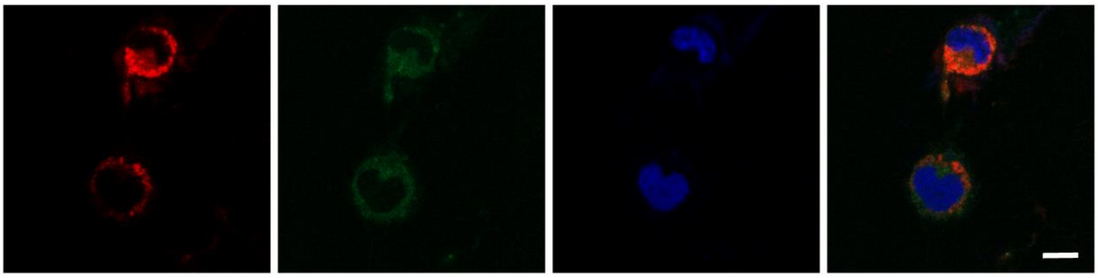

Figure S6. Confocal images showed GVs are located in endosome. The nuclei are stained with Hoechst 33342 (blue) and the endosomes with LysoSensor Green DND-189 (green). Scale bar = 3 μm.

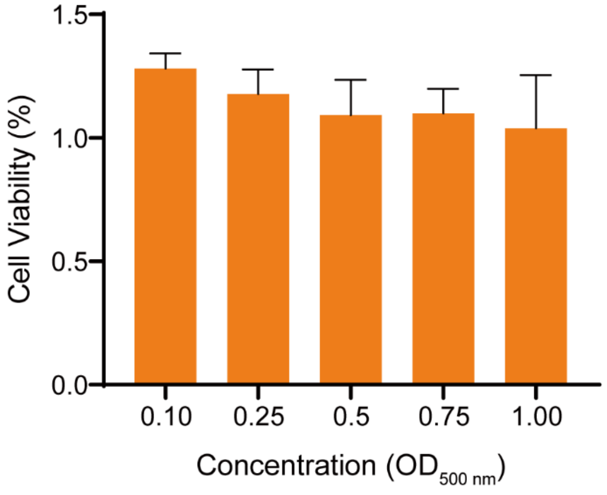

Figure S7. Percentages of viable MSCs after being incubated with GVs at different concentrations. (OD_{500} = 0.1, 0.25, 0.5, 0.75 and 1.0) for 10h (5 replicates).

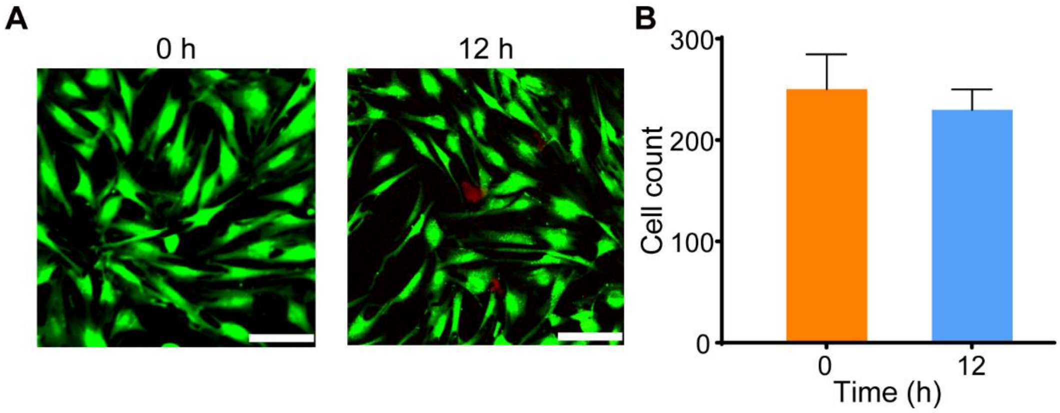

Figure S8. Live and dead cell double staining of MSCs after being incubated with GVs for 0 or 12h. Scale bar = 100 μm (n = 5 fields).

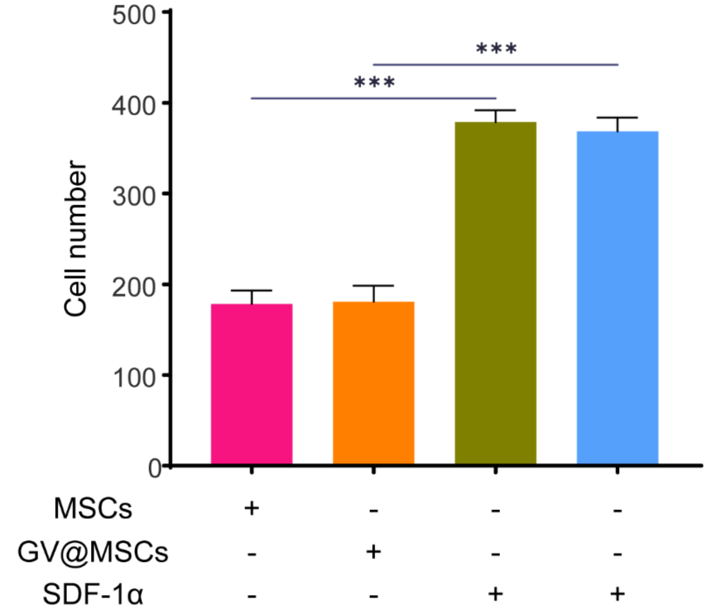

Figure S9. Quantitative analysis of migrating MSCs and GV@MSCs with or without SDF-1α.

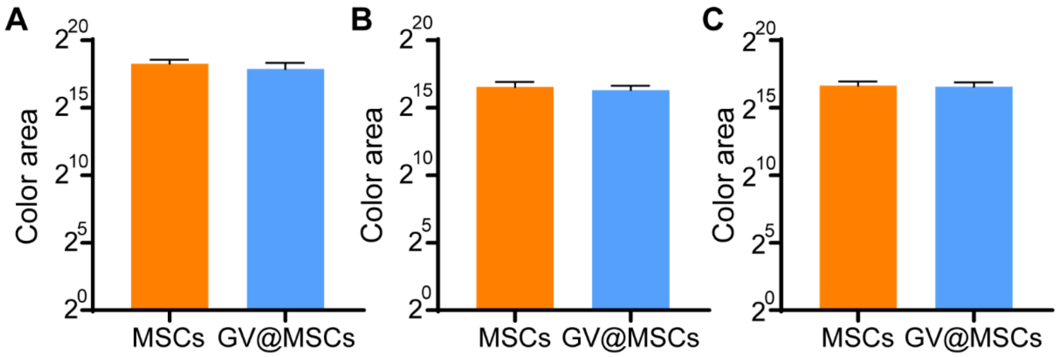

Figure S10. Quantitative analysis of the differentiation capability of MSCs and GV@MSCs by (A) Oil red, (B) Alizarin red and (C) Alixin blue staining.

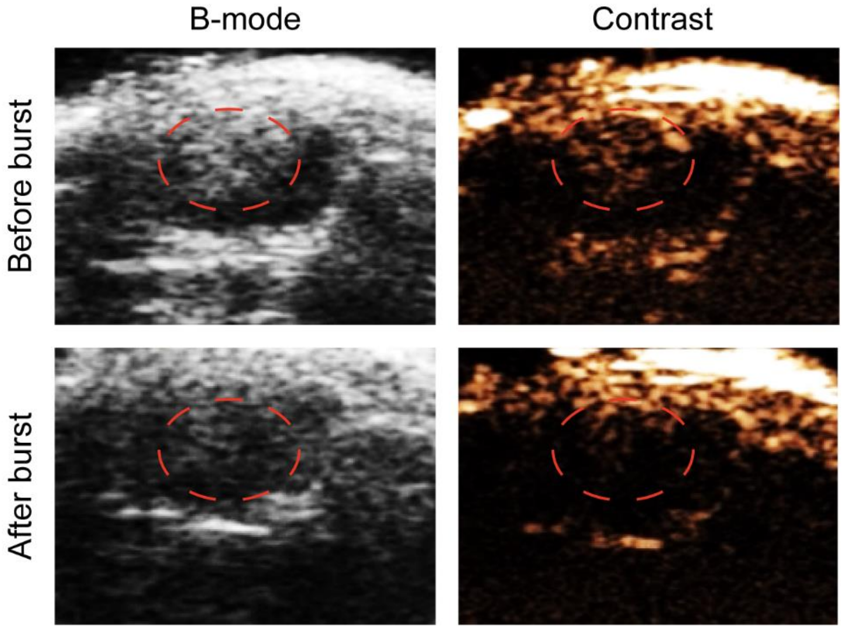

Figure S11. GVs can be burst by ultrasound *in vivo*. Ultrasonic images of GVs before and after destruction by a high-power ultrasound pulse when being subcutaneously injected GV@MSCs into CIA model rats' lateral malleolus joint (1×10^7 cells per rat).
